# Editorial: Neuroimaging in early intervention in psychiatry

**DOI:** 10.3389/fpsyt.2023.1196886

**Published:** 2023-05-05

**Authors:** João Valente Duarte, Sandra Vieira, Nuno Madeira

**Affiliations:** ^1^Coimbra Institute for Biomedical Imaging and Translational Research (CIBIT), Institute for Nuclear Sciences Applied to Health, University of Coimbra, Coimbra, Portugal; ^2^Faculty of Medicine, University of Coimbra, Coimbra, Portugal; ^3^Department of Psychosis Studies, Institute of Psychiatry, Psychology & Neuroscience, King's College London, London, United Kingdom; ^4^Center for Research in Neuropsychology and Cognitive Behavioral Intervention, Faculty of Psychology and Educational Sciences, University of Coimbra, Coimbra, Portugal; ^5^Connectomics Lab, Department of Radiology, Lausanne University Hospital and University of Lausanne (CHUV-UNIL), Lausanne, Switzerland; ^6^Institute of Psychological Medicine, Faculty of Medicine, University of Coimbra, Coimbra, Portugal; ^7^Department of Psychiatry, Centro Hospitalar e Universitário de Coimbra (CHUC), Coimbra, Portugal

**Keywords:** neuroimaging, mental health, diagnosis, early intervention, prognosis

In Psychiatry, understanding disease progression and unpredictable treatment response are still a significant clinical challenge, leading to fruitless therapeutic trials. There is an urgent need to develop biomarkers capable of assisting with real world clinical care and its unmet needs (1). In order to prevent chronic disability and promote long-term recovery, there has been a noticeable shift in the past 20 years toward conducting research during the early stages of illness, when there is an opportunity to intervene (2). This Research Topic brings together original psychiatric neuroimaging studies aimed at investigating possible biomarkers that can assist clinical the decision-making process related to diagnosis, prognosis, and monitoring of individuals who exhibit initial symptoms of an illness or even before the onset of the illness.

Taken collectively, this Research Topic includes a wide range of interesting populations, analytical methods and imaging features, including machine learning to predict the transition of individuals at risk of psychosis; MRI-derived anatomical features of the brain in the early stage of schizophrenia, borderline personality disorder patients with minimal treatment exposure, and individuals with methamphetamine use disorder; the effect of treatment with TMS in functional and structural characteristic patterns in schizophrenia; the effect of antipsychotic treatment in the glutamatergic function in people with first-episode psychosis; and the neural correlates of social exclusion in young individuals with bipolar disorder and their impact on functionality.

After utilizing a rigorous predictive modeling approach, Tavares et al. present compelling evidence that urges a re-evaluation of the predictive potential of structural MRI and genome-wide in identifying the risk of transitioning to psychosis among high-risk individuals. Results show that none of the modalities alone could predict psychosis onset statistically better than chance. However, the authors did not train a multimodal classification model, thus the multivariate nature of neuroimaging combined with genetic and environmental data was not explored, yielding further investigation.

Interestingly, studies after illness onset show anatomical variations that correlate with positive symptomatology, suggesting that such abnormalities may not yet be well-established at the individual level in high-risk individuals. For example, Takahashi, Sasabayashi, Takayanagi et al. showed that altered insular morphology was associated with positive symptoms in early stages and clinical subtypes of schizophrenia. In addition, Cai et al. observed a reduction in gray matter volume in the middle temporal gyrus on both sides and a decrease in cortical thickness in multiple brain regions in individuals with early-onset schizophrenia. In this study, early-onset schizophrenia with genetic risk (first-, second-, or third-degree relatives diagnosed with schizophrenia) showed a different brain structure morphology compared to patients without genetic risk, which indicates that atypical brain structure, particularly in the frontal and temporal lobes, may play a significant role in the pathophysiology of early-onset schizophrenia. This is corroborated by the study of Cobia et al. as it revealed that thalamic shape irregularities were a notable characteristic in both early-onset and late-onset schizophrenia, although more pronounced in the latter group. Additionally, each group displayed distinct brain-behavior patterns (Cobia et al.). Furthermore, the authors proposed that the enduring presence of these irregularities in adult patients with early-onset schizophrenia could signify indicators of disturbed neurodevelopment that are specifically linked to clinical and cognitive aspects of the illness.

Takahashi, Sasabayashi, Velakoulis et al. conducted a second study on this topic, suggesting that neurodevelopmental pathology associated with Heschl's gyrus duplication might be implicated in the neurobiology of early borderline personality disorder patients with minimal treatment exposure, especially for emotional and behavioral control. Interestingly, alterations of gyrification, which are influenced by early neurodevelopment, are further suggested to play a pivotal role in developing mental disorders. Hu et al. showed hypergyrification across multiple brain regions in individuals with methamphetamine use disorder, which was furthermore positively associated with depression and anxiety symptom severity.

While brain structural characteristics in patients with psychiatric illness might constitute biomarkers for early diagnosis, anatomical and functional alterations could also identify target regions for neuromodulation. In one MRI study presented in this Research Topic, Xie et al. investigated the gray matter volume and the seed-based resting-state functional connectivity profile of the nucleus accumbens (Nacc) in individuals with schizophrenia and auditory verbal hallucinations throughout low-frequency repetitive TMS treatment. While the volumetric changes of the NAcc were not impacted, the anomalous functional connectivity patterns of the NAcc in patients before treatment were rectified or reversed after receiving low-frequency repetitive TMS treatment. These FC alterations were linked to symptom and neurocognitive enhancements, indicating that they could serve as a clinical effect biomarker for this treatment approach in individuals with schizophrenia. In a functional study, Roybal et al. investigated brain function with fMRI in youth with bipolar disorder while performing a social exclusion task. Authors found that patients exhibited greater activation in the left fusiform gyrus and significantly decreased functional connectivity of this region with the posterior cingulate/precuneus during social exclusion. Despite having a small sample size, this study proposes that young people with bipolar disorder handle social exclusion by prioritizing basic visual details, while individuals without the disorder rely on prior experiences to comprehend present social interactions. This variance may contribute to the social cognitive challenges faced by those with bipolar disorder, exacerbating symptoms of anxiety and mood disorders.

Lastly, Zahid et al. employed MR spectroscopy in a longitudinal study to examine the impact of antipsychotic therapy on glutamatergic levels in the anterior cingulate cortex (ACC) and to determine whether there was a connection between initial glutamatergic levels and clinical reaction following antipsychotic therapy in individuals experiencing their first episode of psychosis. The authors found no significant impact of antipsychotic treatment on glutamate and glutamate/glutamine levels in the ACC and no correlation between therapeutic outcomes and glutamatergic levels measured prior to antipsychotic administration, indicating null findings. As per this study, it appears that response to treatment is unlikely to be connected to baseline glutamatergic metabolites before antipsychotic therapy, highlighting the need for further investigation of clinically useful biomarkers.

## Author contributions

All authors listed have made a substantial, direct, and intellectual contribution to the work and approved it for publication.
